# Covalent modification of pericardial patches for sustained rapamycin delivery inhibits venous neointimal hyperplasia

**DOI:** 10.1038/srep40142

**Published:** 2017-01-10

**Authors:** Hualong Bai, Jung Seok Lee, Elizabeth Chen, Mo Wang, Ying Xing, Tarek M. Fahmy, Alan Dardik

**Affiliations:** 1 The Vascular Biology and Therapeutics Program and the Department of Surgery, Yale University School of Medicine, New Haven, CT, 06520, USA; 2 Basic Medical College of Zhengzhou University, Henan, China; 3 Department of Vascular Surgery, First Affiliated Hospital of Zhengzhou University, Henan, China; 4 Department of Biomedical Engineering, Yale University, New Haven, CT, 06520, USA; 5 Department of Immunobiology, Yale University School of Medicine, New Haven, CT, 06520, USA; 6 Department of Surgery, VA Connecticut Healthcare System, West Haven, CT 06515, USA.

## Abstract

Prosthetic grafts and patches are commonly used in cardiovascular surgery, however neointimal hyperplasia remains a significant concern, especially under low flow conditions. We hypothesized that delivery of rapamycin from nanoparticles (NP) covalently attached to patches allows sustained site-specific delivery of therapeutic agents targeted to inhibit localized neointimal hyperplasia. NP were covalently linked to pericardial patches using EDC/NHS chemistry and could deliver at least 360 ng rapamycin per patch without detectable rapamycin in serum; nanoparticles were detectable in the liver, kidney and spleen but no other sites within 24 hours. In a rat venous patch angioplasty model, control patches developed robust neointimal hyperplasia on the patch luminal surface characterized by Eph-B4-positive endothelium and underlying SMC and infiltrating cells such as macrophages and leukocytes. Patches delivering rapamycin developed less neointimal hyperplasia, less smooth muscle cell proliferation, and had fewer infiltrating cells but retained endothelialization. NP covalently linked to pericardial patches are a novel composite delivery system that allows sustained site-specific delivery of therapeutics; NP delivering rapamycin inhibit patch neointimal hyperplasia. NP linked to patches may represent a next generation of tissue engineered cardiovascular implants.

Neointimal hyperplasia, the proliferation and migration of smooth muscle cells with deposition of extracellular matrix, remains a common problem after vascular surgery that limits the durability of interventions; neointimal hyperplasia is currently the leading cause of vascular graft failure and restenosis after arterial interventions^1^,^2^. The disappointing failure of large, multicenter randomized trials to eliminate neointimal hyperplasia show that new approaches are needed^3^,^4^. Current technology has led to the clinical popularity of drug-eluting stents that deliver rapamycin and paclitaxel to inhibit neointimal hyperplasia, and serve as a model of enhancing primary technologies with additional drug-delivery mechanisms^5^,^6^,^7^,^8^. However, drug-eluting stents are useful for endovascular technologies but do not address neointimal hyperplasia that forms after traditional open surgical procedures.

Nanotechnology may allow further advances in drug delivery; nanoparticles (NP) can be made from many materials and can be used to encapsulate many types of therapeutic agents. Poly (lactic-co-glycolic acid) (PLGA) is approved for use in patients and is one of the most effective biodegradable polymeric NP in current use^9^,^10^. In a rabbit femoral artery injury model, a 5 minute infusion of a rapamycin nanoparticle emulsion into the injured vessel lumen was sufficient to reduce neointimal hyperplasia by 52% at 2 weeks^11^. Similarly, in the rat balloon injury model, Reddy *et al*. reported that localized infusion of a suspension of rapamycin-containing nanoparticles to the endothelium for 15 minutes after the balloon injury was sufficient to allow sustained tissue levels of rapamycin as well as significant inhibition of neointimal hyperplasia at 3 weeks^12^.

Rapamycin-containing NP have also been used to treat vein grafts in the arterial environment. In the rabbit vein graft model, pretreatment of vein grafts with a single intraluminal perfusion of carbopol-encapsulated rapamycin-loaded PLGA nanoparticles allowed retention of rapamycin for 28 days, reduced cell proliferation, and inhibited neointimal hyperplasia^13^. Similarly, in a rat vein graft model, NP-containing rapamycin showed sustained release and inhibited vein graft thickening, without inhibiting endothelial cell proliferation^14^. Given the promise of NP-mediated delivery to inhibit neointimal hyperplasia, an optimal method to incorporate and deliver NP is a much sought after goal for translational applications.

Pericardial patches, either bovine or porcine, are commonly used by surgeons to close blood vessels during open cardiovascular surgery^15^,^16^. The pericardial patch is composed of wavy bundles of collagen, allowing ease of handling and suturing in the operating room as well as biocompatibility. Similar in concept to the addition of drug-eluting capability to bare metal stents to create drug-eluting stents, we hypothesized that NP-containing therapeutics can be covalently conjugated to pericardial patches as a next-generation tissue engineered drug delivery platform. More specifically, we hypothesized that implantation of pericardial patches, with conjugated NP-containing therapeutics, would allow site-specific delivery of therapeutic agents ideally targeted to inhibit localized neointimal hyperplasia that forms on these implanted patches. Accordingly, we developed a venous model of patch angioplasty that shows stable and aggressive neointimal hyperplasia compared to that which develops in arteries^17^, based upon our previous experience with arterial patch angioplasty^18^. We conjugated rapamycin-containing NP (NP-rapamycin) to bovine pericardial patches, and determined the ability of the localized drug to inhibit neointimal hyperplasia that develops on the pericardial patch. In addition, we tested the organ distribution of NP after implantation into the venous system to determine if NP conjugated to pericardial patches can be found systemically, potentially producing off-target effects that might limit local delivery.

## Results

### Nanoparticle-mediated sustained rapamycin release from pericardial patches inhibits SMC proliferation and mTOR phosphorylation *in vitro*

**Figure 1:**
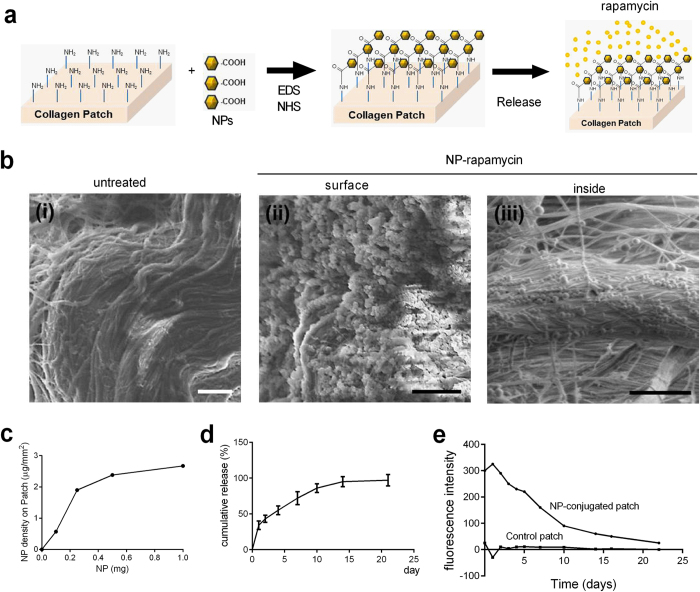
Nanoparticles (NP) conjugated to the pericardial patch. (**a**) Schematic illustration of preparation of a collagen patch chemically conjugated with NP. Amines on the patch were coupled with carboxyl groups of the NP using the EDC-NHS chemistry. The NP release rapamycin over time. (**b**) Representative photomicrographs of non-treated collagen patch (i), and patches conjugated with NP loaded with rapamycin (NP-rapamycin) (surface (ii) and inside (iii)); scale bars, 2 μm. (**c**) Curve showing effect of differing NP amount fed into the coupling reaction on NP-patch conjugation density. (**d**) Elution curve showing rapamycin release from NP-rapamycin over 21 days. (**e**) Relative intensity curve showing NP-rhodamine release from patches over 21 days.

Since rapamycin inhibits neointimal hyperplasia in both arteries and vein grafts^5^, we determined whether patches could be used for site-specific delivery of rapamycin in veins. We encapsulated rapamycin within nanoparticles (NP-rapamycin; 30 ng rapamycin/μg nanoparticle; mean NP diameter 369.9 nm; mean zeta-potential −37.8 ± 2.9 mV; rapamycin encapsulation efficiency 85.7%) and conjugated them to pericardial patches (Fig. 1a); scanning electron microscopy confirmed the presence of NP-rapamycin on the surface as well as inside of the patches, within the interstices of the collagen fibers (Fig. 1b). The density of nanoparticles conjugated to the patch was linear at low numbers but was saturated at higher numbers of nanoparticles (Fig. 1c). Since nanoparticles were conjugated to patches at a density of approximately 2.67 μg/mm^2^, and the implanted patch was approximately 3 × 1.5 × 0.6 mm (length × width × height), there were at least approximately 360 ng of rapamycin in 12 μg of nanoparticles. Rapamycin release from the NP-rapamycin-conjugated patches plateaued by day 15 *in vitro*, suggesting the ability to deliver the drug for at least 2 weeks (Fig. 1d). NP were released from patches over approximately 3 weeks *in vitro* (Fig. 1e).

**Figure 2:**
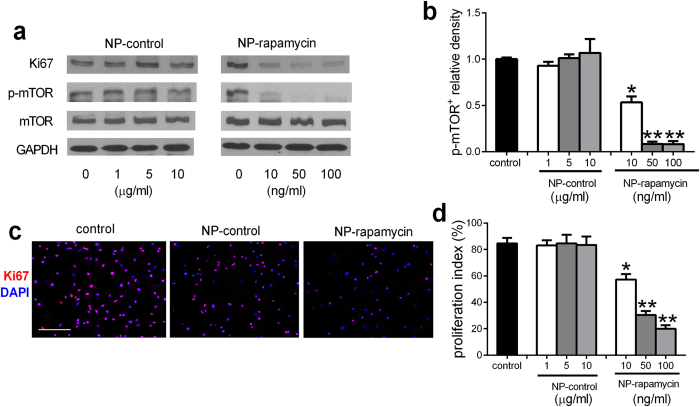
NP-rapamycin inhibits SMC proliferation and p-mTOR expression *in vitro*. (**a**) Representative Western blot showing expression of Ki67, p-mTOR, mTOR and GAPDH in human aorta SMC treated with NP-control or NP-rapamycin for 24 h; n = 3. (**b**) Bar graph showing the relative density of p-mTOR expression. p < 0.0001, one-way ANOVA; *p < 0.0001, vs. control and NP-control; **p < 0.02, vs. 10 ng/ml; post hoc. n = 3. (**c**) Immunofluorescence analysis of human aorta SMC treated with control, NP-control (1 μg/ml) and NP-rapamycin (10 ng/ml) for 24 h. Merge of Ki67 (red) and DAPI (blue); scale bar, 100 μm. n = 3. (**d**) Bar graph shows the proliferation index in human aorta SMC treated with control, NP-control and NP-rapamycin for 24 h; mean of 4 high power fields; p < 0.0001, one-way ANOVA; *p < 0.0001, vs. control and NP-control; **p < 0.0001, vs. 10 ng/ml; post hoc. n = 3. Full-size blots are presented in Supplementary Figure 5.

Since rapamycin inhibits SMC proliferation, we examined the proliferative response of SMC to NP-rapamycin *in vitro*. SMC treated with control NP showed no change in Ki67 expression or phosphorylation of mTOR, whereas SMC treated with NP-rapamycin showed diminished Ki67 expression and phosphorylation of mTOR (Fig. 2). These results are consistent with NP-rapamycin inhibiting SMC proliferation *in vitro*, a similar effect as seen *in vivo*, e.g. rapamycin delivered from NP-conjugated patches remains biologically active.

### Nanoparticle-mediated rapamycin release from pericardial patches inhibits localized neointimal hyperplasia

**Figure 3:**
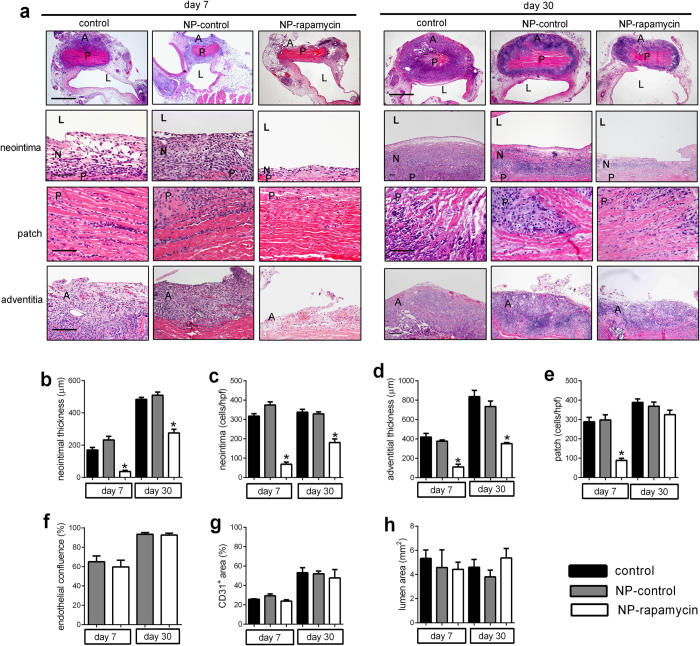
Rapamycin-eluting nanoparticles conjugated to pericardial patches reduce neointimal and adventitia thickness after patch venoplasty. (**a**) Representative photomicrographs of control patches, NP-control patches or NP-rapamycin patches explanted at day 7 and day 30 stained with H&E; first row, low power, scale bar, 1 mm; second row showing the neointima, scale bar, 100 μm; third row showing the patch, scale bar, 100 μm; fourth row showing the adventitia, scale bar, 200 μm; L, lumen; N, neointima; P, patch; A, adventitia; n = 4–8. (**b**) Bar graph showing the neointimal thickness at day 7 and day 30; *p < 0.001, one-way ANOVA; vs. control and NP-control. n = 4–8. (**c**) Bar graph showing neointimal cell number at day 7 and day 30; *p < 0.001, one-way ANOVA; vs. control and NP-control. n = 4–8. (**d**) Bar graph showing the adventitia thickness at day 7 and day 30; *p < 0.002, vs. control and NP-control. n = 4–8. (**e**) Bar graph showing patch cell number at day 7 and day 30; p = 0.0007, one-way ANOVA; vs. control and NP-control. n = 4–8. (**f**) Bar graph shows neointimal endothelial confluence at day 7 and day 30; p > 0.5, t-test. n = 4–8. (**g**) Bar graph shows neointimal CD31 positive cell area at day 7 and day 30; p > 0.1, one-way ANOVA. n = 3. (**h**) Bar graph shows vessel luminal area at day 7 and day 30; p > 0.2, one-way ANOVA. n = 4–8.

To determine the effects of NP-rapamycin-conjugated pericardial patches on patch-associated neointimal hyperplasia, NP-rapamycin-conjugated pericardial patches were implanted in veins and the amount of neointimal hyperplasia was compared to patches without any NP (control) as well as to patches conjugated with NP but not containing any rapamycin (NP-control). At day 7, a thick adventitia covered the patch in the control and NP-control group, whereas a very thin adventitia covered the patch in the NP-rapamycin group (Fig. 3a, first row, fourth row; Fig. 3d; Supplemental Figure 1a); the adventitia was thick in all groups by day 30 (Supplemental Figure 1a). After 7 days there was significantly less neointima formed on NP-rapamycin patches compared to both control and NP-control patches (Fig. 3a, second row; Fig. 3b); after 30 days, the neointima in the NP-rapamycin patches was also thinner than the control and NP-control patches (Fig. 3a, second row; Fig. 3b). Patches conjugated with NP-rapamycin showed fewer neointimal cells at both day 7 and day 30 (Fig. 3a, second row; Fig. 3c), as well as fewer cells in the body of the NP-rapamycin patches (Fig. 3a, third row; Fig. 3e). There was also reduced thickness of the adventitia on the NP-rapamycin patches (Fig. 3a, first row, fourth row; Fig. 3d), without any difference in vein lumen area (Fig. 3a, first row; Fig. 3h).

CD31-positive cells were present at the neointimal surface in both NP-control and NP-rapamycin patches (Supplemental Figure 1b), with increasing endothelial coverage over time in both groups (Fig. 3f). En-face staining confirmed the presence of CD31 positive cells on the neointimal surface of all patches, with no difference in endothelial area at either day 7 or day 30 (Fig. 3g, Supplemental Figure 1c). There were also a similar number of cells that were both CD34- and VEGFR2-positive, both CD34- and Eph-B4-positive, as well as CD31- and Eph-B4-positive in NP-rapamycin and NP-control patches, consistent with a similar number of EPC, venous EPC, as well as mature venous endothelial cells, respectively (Supplemental Figure 1b).

**Figure 4:**
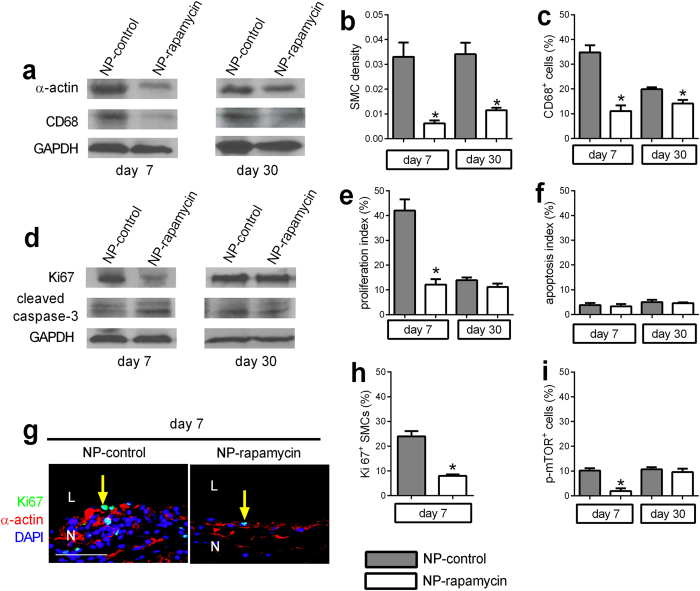
NP-rapamycin inhibits SMC and macrophage infiltration and proliferation. (**a**) Representative Western blot showing expression of α-actin, CD68 and GAPDH in NP-control or NP-rapamycin-eluting patch neointimas at day 7 or day 30; n = 3. (**b**) Bar graph showing SMC density. *p < 0.02, t-test; n = 4; (**c**) Bar graph showing percentage of CD68 -positive cells at day 7 or day 30. *p < 0.03, t-test. n = 4. (**d**) Representative Western blot showing expression of Ki67, cleaved caspase-3 and GAPDH in NP-control or NP-rapamycin-eluting patches at day 7 or day 30; n = 4. (**e**) Bar graph showing proliferation index at day 7 or day 30. *p = 0.0039, t-test. n = 4. (**f**) Bar graph showing apoptosis index at day 7 or day 30. n = 4. (**g**) Immunofluorescence analysis of the neointima in NP-control and NP-rapamycin eluting patches (day 7). Merge of Ki67 (green), α-actin (red) and DAPI (blue); L, lumen; N; neointima; scale bar, 50 μm. n = 4. (**h**) Bar graph shows the percentage of Ki67 and α-actin dually-positive cells in the neointima (day 7) in the NP-rapamycin vs. NP-control group; mean of 4 high power fields per patch; *p = 0.0018, t-test; n = 4. (**i**) Bar graph showing percentage of p-mTOR positive cells at day 7 and day 30. *p = 0.0046, t-test; n = 4. Full-size blots are presented in Supplementary Figure 5.

There was decreased α-actin expression in NP-rapamycin patches at both day 7 and day 30 compared to NP-control patches (Fig. 4a), with decreased SMC density in NP-rapamycin patches (Fig. 4b; Supplemental Figure 2a). There was also decreased expression of CD68 and fewer CD68-positive cells in NP-rapamycin patches (day 7) (Fig. 4a,c; Supplemental Figure 2a), but without any reduction in number of M2 type macrophages in NP-rapamycin patches (Supplemental Figure 2b–d). NP-rapamycin patches had less Ki67 expression and fewer proliferating cells (day 7) compared to NP-control patches (Fig. 4d,e; Supplemental Figure 2a). Cleaved caspase-3 expression and apoptotic cells were similar in NP-rapamycin and NP-control patches at both days 7 and 30 (Fig. 4d,f; Supplemental Figure 2a). NP-rapamycin patches showed fewer proliferating SMC compared to those in NP-control patches (day 7) (Fig. 4g,h). There were also fewer p-mTOR-positive cells in NP-rapamycin patches (day 7) (Fig. 4i; Supplemental Figure 2a).

### Specific distribution of nanoparticles after implantation *in vivo*

**Figure 5:**
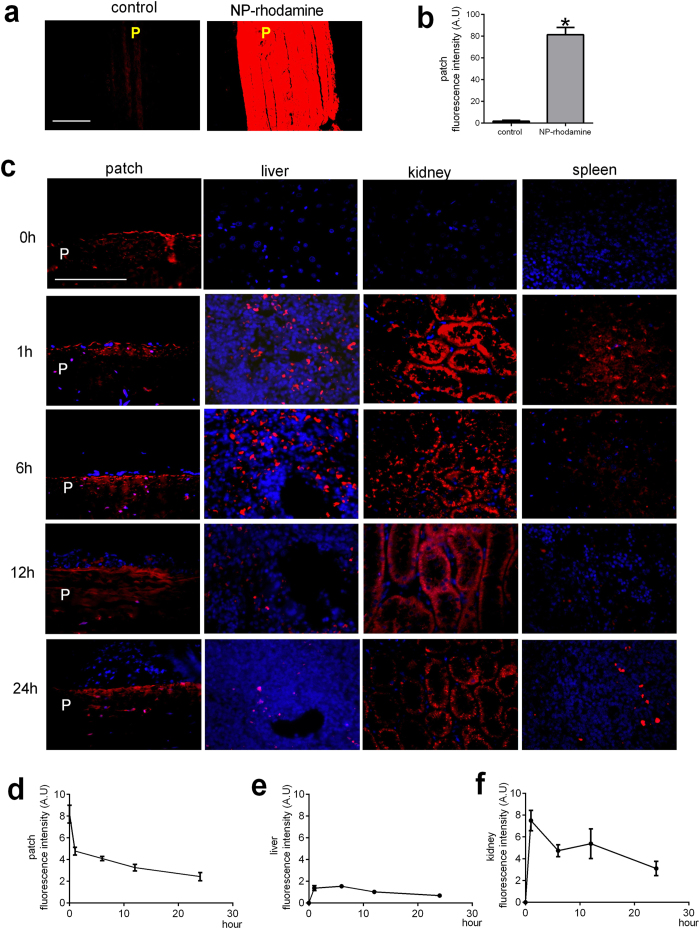
Nanoparticle trafficking in rat tissues. (**a**) Patch without and with nanoparticle conjugation; nanoparticles contain rhodamine; scale bar, 100 μm; p, patch; n = 3. (**b**) Immunofluorescence intensity of nanoparticles; *p = 0.0003, t-test; n = 3. (**c**) Time course of nanoparticle fluorescence on the patch surface, liver, kidney, and spleen, 0–24 hours after implantation; scale bar, 100 μm; p, patch; n = 3. (**d**) Line graph shows immunofluorescence intensity of nanoparticles on the patch luminal surface; n = 3. (**e**) Line graph shows immunofluorescence intensity of nanoparticles in the liver; n = 3. (**f**) Line graph shows immunofluorescence intensity of nanoparticles in the kidney; n = 3.

To determine the distribution of nanoparticles after implantation, patches were conjugated with nanoparticles containing rhodamine and then implanted into the IVC; after NP-rhodamine conjugation, patches had strong immunofluorescence compared to control patches (Fig. 5a,b). NP-rhodamine patches retained their immunofluorescence for at least 24 hr after implantation (Fig. 5c). NP-rhodamine was also detectable in the rat liver, kidney and spleen (Fig. 5c–f), with very little NP-rhodamine in the lung, heart, skeletal muscle, brain or aorta (Supplemental Figure 3a).

Rapamycin was not detectible in serum on days 1, 3 and 7 after implantation of NP-rapamycin patches (data not shown; ELISA detection threshold 5 ng/ml). Similarly, there was no decrease in proliferation in the lungs, liver, kidney, or spleen of rats that had NP-rapamycin patches compared to control or NP-control patches (Supplemental Figure 3b,c; Supplemental Figure 4). There was also no increase in apoptosis in the lungs, liver, kidney, or spleen of rats that had NP-rapamycin patches compared to control or NP-control patches (Supplemental Figure 3b,d; Supplemental Figure 4).

## Discussion

In summary, we developed a next-generation tissue engineered drug delivery platform by covalently linking rapamycin-containing NP to a bovine pericardial patch commonly used in cardiovascular surgery. Using a venous patch angioplasty model that forms aggressive neointimal hyperplasia, patches with rapamycin-containing NP show reduced thickening. This modified patch is a novel composite delivery system that allows sustained site-specific delivery of therapeutics and may represent a next generation of tissue engineered implants.

Pericardial patches, either bovine or porcine, are commonly used by surgeons to close blood vessels during open cardiovascular surgery^15^,^16^. Recent studies from our laboratory showed that the pericardial patch may be a unique niche microenvironment that attracts endothelial progenitor cells after implantation, promoting incorporation into the host vessel that may be responsible for long term durability and clinical success^18^,^19^. Although open venous surgery is performed less frequently compared to open arterial surgery, patches are also used in venous surgery^20^, as venous procedures are frequently complicated by aggressive neointimal hyperplasia and restenosis, possibly due to the lower shear stress in the venous system^21^,^22^,^23^,^24^,^25^. In addition, patients with severe vascular disease may develop spontaneous venous neointimal hyperplasia^26^, placing them at additional risk after conventional venous interventions. The high rates of neointimal hyperplasia and restenosis after venous interventions shows the persistent clinical need for improved techniques or devices to treat patients with venous stenosis, and particularly suggest the utility of patch venoplasty^17^.

We covalently linked NP to a pericardial patch; since these patches are primarily collagen fibers, the NP are conjugated throughout the patch in addition to the patch surface (Fig. 1), facilitating a localized and increased concentration profile of the released drug. In previous work, Labowsky *et al*. described an in silico model demonstrating how the nature and profile of this concentration gradient near a cell surface develops over time and space, although the upper limit of drug delivery is not known^27^. Covalent linkage of the NP allows delivery of therapeutic agents directly into the circulatory system with minimal shedding of the NP into the systemic circulation and most organs, although some NP can be found in the liver, kidney and spleen. The use of rapamycin-containing NP to inhibit neointimal hyperplasia has been reported^14^, but these studies used directly injected NP. We show that a commonly used patch can be used as a next-generation delivery system for these agents to deliver drugs in a sustained fashion at a specific site. In this study, the amount of drug deliverable on the patch depends on both the numbers of NP used in the coupling reaction (Fig. 1c), the porosity of the patch (Fig. 1b), as well as the size of the implanted patch. However, it is likely that other applications with other NP-drugs will require different NP densities that would need to be optimized as a function of the reagent delivered and/or the conjugation reaction conditions.

We capitalize upon a novel venous model of patch angioplasty^17^. First, placement of the patches in the venous system allows detection of off-target delivery of therapeutics, especially to the liver and lungs. Second, modification of the NP or the linkage systems may allow different kinetic profiles of drug delivery, potentially allowing targeted delivery to more distal locations. Third, the more aggressive development of neointimal hyperplasia in venous systems provides a robust test of the delivery system^17^,^28^,^29^. Lastly, the surgical patch angioplasty method may limit restenosis after drug delivery is reduced below therapeutic levels, e.g. after the drug is exhausted, since the patched site has less tendency for form restenosis, thereby increasing the translational potential of patch angioplasty^30^,^31^,^32^,^33^.

In conclusion, we report the construction and therapeutic application of a next-generation drug delivery system using pericardial patches as a novel tissue engineering platform. Conjugation of NP containing therapeutics to collagen scaffolds represents a step forward in delivering therapeutics, both into the vascular system, and potentially downstream into target organs.

## Methods

### Synthesis and characterization of rapamycin-loaded nanoparticles

We chose the Poly(lactic-co-glycolic acid) (PLGA) polymer as the core material for nanoparticles (NP), due to its well-established preparation methodologies and established use in humans. NP were made encapsulating rhodamine for biodistribution studies or rapamycin for the present application. NP were prepared using an established emulsion method^14^,^34^,^35^,^36^. Carboxylated PLGA (100 mg) and rhodamine (1 or 10 mg) or rapamycin (5 mg) were dissolved in chloroform, and then added drop-wise to 5% polyvinyl alcohol (PVA). The mixture was sonicated three times and then added to 0.2% PVA solution. The solvent was evaporated for 2 h while stirring and the PLGA particles were centrifuged before lyophilization as previously described.

The size and polydispersity Index (PDI) of the nanoparticles were measured using a Zetasizer (Malvern, Westborough, MA). Light scattering was measured by back-scattering at a detection angle of 173° and a wavelength of 532 nm; the hydrodynamic radius was calculated using the Stokes-Einstein equation as previously described^34^,^35^,^36^. To measure the loading of rhodamine, nanoparticles were dissolved in DMSO and analyzed in a plate reader (λex 575 nm; λem 605 nm). For rapamycin, NaOH (1 M) was added in DMSO samples and absorption at 400 nm was measured.

### Covalent modification of patches with rapamycin-loaded NP

Pericardial patches were trimmed to 7 mm × 5 mm pieces and ethyl(dimethylaminopropyl) carbodiimide/*N*-hydroxysuccinimide (EDC/NHS) chemistry was then used to conjugate the NP to the patch. EDC (20 μmol/mL) and NHS (10 μmol/mL) were dissolved in MES solution (0.1 M; pH 5) for the conjugation. Carboxyl groups on the NP were activated using EDC (1 mL) for 30 min and substituted with NHS by adding NHS (1 mL) for 30 min. The solution pH was raised from 5 to 8 using NaOH (1 M; 200 μL). Patches were then placed into solution and incubated at 37 °C overnight.

### Assessment of rapamycin and NP release from patches

Patches conjugated with NP containing rapamycin (NP-rapamycin) were incubated in PBS at 37 °C. The supernatant of each sample was collected and absorption (400 nm) was analyzed at each time point using a SpectraMax plate reader (Molecular Devices, Sunnyvale, CA). Patches conjugated with NP containing rhodamine (NP-rhodamine) were incubated in PBS at 37 °C; control patches were incubated in PBS containing free rhodamine. At various time points patches were washed in PBS and their fluorescence intensity was measured.

### Assessment of patch-NP topology using scanning electron microscopy (SEM)

Scanning electron microscopy (SEM) was performed using a Hitachi S-4800 High Resolution SEM (Hitachi High Technologies Inc., Tokyo, Japan). Collagen patches before and after conjugation of NPs were lyophilized and mounted on the aluminum sample holder to be sputter-coated with chromium. The patches were observed with an accelerating voltage of 15 kV at a working distance of 4 mm.

### Serum rapamycin assay

Following explantation of NP-rapamycin conjugated patches on days 1, 3, or 7 (n = 9), whole blood was collected from the IVC (4 ml) and transferred to a BD Microtainer^®^ MAP Microtube with K2EDTA (1.0 mg). Samples were analyzed using liquid chromatography-tandem mass spectrometry (LC-MS/MS)(Waters Acquity; Department of Laboratory Medicine, Yale-New Haven Hospital, New Haven, CT).

### Human smooth muscle cell culture

Human smooth muscle cells (SMC), passages 6–8, were cultured in endothelial basal medium 2, supplemented with endothelial cell growth media-2 MV SingleQuot Kit Supplement & Growth Factors (Lonza), 20% fetal bovine serum, penicillin/streptomycin, and L-glutamine (Corning Life Sciences). At approximately 60% confluence, NP and NP-rapamycin were added to the cells. Cells were generously provided by Dr. Mingzhu Yin, Pathology Department, Yale School of Medicine, New Haven, CT.

### Animal Model

Male Wistar rats (6–8 week old) were used for patch implantation (n = 111). Microsurgical procedures were performed aseptically in a dedicated facility using a dissecting microscope (Leica MZ 95, Germany). Anesthesia was given via isoflurane inhalation. A midline incision was made in the abdomen, and the infrarenal vena cava (IVC) was exposed. The site of patch implantation was approximately 2 mm below the level of the origin of the renal veins; the IVC was dissected free at this site, and all lumbar veins at this level were ligated and divided using 6–0 nylon sutures. The infrarenal IVC was clamped and a longitudinal 3 mm venotomy was made on the anterior wall of the IVC. The venotomy was closed with a pericardial patch (3 mm × 1.5 mm × 0.6 mm; Xenosure; LeMaitre Vascular, Burlington, MA) using interrupted 10–0 nylon sutures. After completion of the venoplasty closure, the clamps were removed to vent the patch and then restore blood flow in the IVC. The abdomen was closed using 5-0 Dacron sutures. Rats were sacrificed on postoperative 0 h, 1 h, 6 h, 12 h, 24 h, days 7, or 30 and the patches and organs explanted for analysis. No immunosuppressive agents, antibiotics or heparin were given at any time.

### Histology

Rats were anesthetized with isoflurane inhalation, and tissues were fixed by transcardial perfusion of phosphate buffered saline (PBS) followed by 10% formalin. Tissue was removed and fixed overnight in 10% formalin followed by a 24-hour immersion in 70 percent alcohol. Tissue was then embedded in paraffin and sectioned (5 μm thickness). Tissue sections were de-paraffinized and stained with hematoxylin and eosin.

### Immunohistochemistry

Tissue sections were de-paraffinized and then incubated using primary antibodies overnight at 4 °C. After overnight incubation, the sections were incubated with EnVision reagents for 1 h at room temperature and treated with Dako Liquid DAB Substrate Chromogen System (Dako). Finally, the sections were counterstained with Dako Mayer’s Hematoxylin.

### Immunofluorescence

Tissue sections with rhodamine were de-paraffinized and examined under the immunofluorescence microscope directly. Otherwise, tissue sections were de-paraffinized and then incubated with primary antibodies overnight at 4 °C. To visualize and quantify cells, sections were stained with the fluorescent dye 4′,6-diamidino-2-phenylindole (DAPI; Invitrogen) to mark cellular nuclei.

### Western blot

Pericardial patches were carefully harvested and removed from surrounding tissue; the neointima was carefully dissected free from the patch and snap frozen in liquid nitrogen. Samples were crushed and mixed with buffer including protease inhibitors (Roche, Complete Mini 12108700) prior to sonication (5 sec) and centrifugation (135000 rpm, 15 min). Equal amounts of protein from each experimental group were loaded for SDS-PAGE, followed by Western blot analysis with signals detected using the ECL detection reagent. Patches were analyzed individually, without combination of samples.

### Primary and secondary antibodies

Primary antibodies included: anti-α-actin (Abcam, ab5694; IHC and IF, 1:100; WB, 1:1000); Dako actin (Smooth Muscle) Clone 1A4; anti-cleaved Caspase-3 (Cell Signaling #9661; IHC, 1:50; WB, 1:1000); anti-CD31 (Abcam, ab28364; IHC and IF, 1:50); anti-CD34 (R&D, AF4117; IF, 1:100; WB, 1:1000); anti-CD68 (ED1; Abcam, ab31630; IHC, 1:200; WB, 1:1000); anti-Eph-B4 (Santa Cruz, sc-5536; IF, 1:50); anti-Ephrin-B2 (Novus, NBP1-48610; IHC, 1:50); anti-GAPDH (Cell Signaling, 14C10; WB, 1:2000); anti-IL-10 (Abcam, ab9969; IF, 1:100); anti-Ki67 (Abcam, ab15580; IHC and IF, 1:100; WB, 1:1000); anti-phospho-mTOR (Cell Signaling, #2971; IHC, 1,50; WB,1:1000); anti-mTOR (Cell Signaling, #2972; WB, 1:1000); anti-transglutaminase 2 (TGM2; #37557; IF, 1:100); anti-VEGFR2 (ABCAM, ab2349; IF, 1:100; WB,1:1000); Secondary antibodies used for IF were: donkey anti-goat Alexa-Fluor-488, donkey anti-rabbit Alexa-Fluor-488, donkey anti-rabbit Alexa-Fluor-568, donkey anti-mouse Alexa-Fluor-568 and chicken anti-mouse Alexa-Fluor-488 conjugated antibodies from Invitrogen (1:1000). For IHC, sections were incubated with EnVision reagents for 1 h at room temperature and treated with Dako Liquid DAB + Substrate Chromogen System (Dako). Finally, the sections were counterstained with Mayer’s hematoxylin.

### Enface staining

Rats were anesthetized with isoflurane inhalation, and blood was flushed with phosphate buffered saline (PBS) by transcardial perfusion. The patch and IVC were explanted and fixed with 4% PFA (15 min) prior to incubation with primary antibodies overnight at 4 °C. The patch and IVC were then incubated with EnVision reagents for 1 h at room temperature and treated with Dako Liquid DAB Substrate. Samples were directly visualized with a dissecting microscope under 63× magnification.

### Statistical analysis

Data are expressed as the mean ± SEM. Statistical significance for these analyses was determined by ANOVA and t-test. P-values less than 0.05 were considered significant. The mean integrated optical density (IOD) of the immunohistochemistry in the neointima was analyzed using Image-Pro Plus 6.0 software (Media Cybernetics; Rockville, MD). The rhodamine immunofluorescence density was analyzed using imageJ (National Institutes of Health).

### Study Approval

All experiments were approved by the Institutional Animal Care and Use Committee at the Yale University School of Medicine. All experiments were carried out in accordance with the approved guidelines.

## Additional Information

**How to cite this article**: Bai, H. *et al*. Covalent modification of pericardial patches for sustained rapamycin delivery inhibits venous neointimal hyperplasia. *Sci. Rep.* **7**, 40142; doi: 10.1038/srep40142 (2017).

**Publisher's note:** Springer Nature remains neutral with regard to jurisdictional claims in published maps and institutional affiliations.

## Supplementary Material

Supplementary Figures
