# Supplementary material for: Covalent modification of pericardial patches for sustained rapamycin delivery inhibits venous neointimal hyperplasia
Source: Sci Rep. 2017 Jan 10;7:40142. doi: 10.1038/srep40142 (PMC5223139; doi:10.1038/srep40142)

Covalent modification of pericardial patches for sustained rapamycin delivery inhibits venous neointimal hyperplasia

Hualong Bai,[a,b,c] Jung Seok Lee,[d] Elizabeth Chen,[a] Mo Wang,[a]

Ying Xing,[b] Tarek M. Fahmy,[d,e] Alan Dardik[a,f]

a. The Vascular Biology and Therapeutics Program and the Department of Surgery, Yale University School of Medicine, New Haven, CT, 06520
b. Basic Medical College of Zhengzhou University, Henan, China
c. Department of Vascular Surgery, First Affiliated Hospital of Zhengzhou University, Henan, China
d. Department of Biomedical Engineering, Yale University, New Haven, CT, 06520
e. Department of Immunobiology, Yale University School of Medicine, New Haven, CT, 06520
f. Department of Surgery; VA Connecticut Healthcare System, West Haven, CT 06515

Supplemental Figure 1. Rapamycin-eluting nanoparticles conjugated to pericardial patches reduce neointimal thickness after patch venoplasty. a) Representative photomicrographs of the adventitial surface (black arrow) of control patches, NP-control patches or NP-rapamycin patches (day 7 and day 30); A, aorta; IVC, inferior vena cava; ruler denotes 1mm, n=4. **b)** Immunohistochemical and immunofluorescence analysis of the neointima at day 7 and day 30; first row, immunohistochemical staining of CD31, second row, merge of CD34 (green), VEGFR2 (red) and DAPI (blue); third row, merge of CD34 (green), Eph-B4 (red) and DAPI (blue); bottom row, merge of CD31 (green), Eph-B4 (red) and DAPI (blue). L, lumen; N, neointima; scale bar, 50 µm. Yellow arrows show colocalizing cells. n=4-8. **c)** Luminal view of the neointima at day 7 and day 30 in the control, NP-control and NP-rapamycin patches; en face staining for CD31; scale bar, 1mm; n=3. Yellow arrows show the positive cells**.**

Supplemental Figure 2. Cell composition after patch venoplasty, without and with rapamycin-eluting nanoparticles. a) Immunohistochemical analysis for α-actin, CD68, Ki67, cleaved caspase-3, and phospho-mTOR of the neointima in NP-control and NP-rapamycin eluting patches at day 7 (upper row) and day 30 (lower row). L, IVC lumen; Scale bar, 50 µm; n=4-8. Yellow arrows show the positive cells. **b)** Representative photomicrographs of the neointima in control, NP-control or NP-rapamycin patches at day 7 (upper row) or day 30 (lower row); merge of TGM2 (red), CD68 (green) and DAPI (blue); merge of IL-10 (red), CD68 (green) and DAPI (blue); L, lumen; N, neointima; P, patch; scale bar, 100 µm; n=4-6. Yellow arrows show colocalization. **c)** Bar graph shows

the number of TGM2 positive macrophages per high power field; p=0.2016, one-way ANOVA, n=4-6. **d)** Bar graph shows the number of IL-10 positive macrophages per high power field; p=0.7025, one-way ANOVA, n=4-6.

Supplemental Figure 3: Nanoparticle trafficking in rat tissues and influence of nano-particle rapamycin on rat lung. **a)** Time course of nanoparticle fluorescence on the lung, heart, skeletal muscle, brain and aorta 0-24 hours after implantation; L, aorta lumen; scale bar, 100µm; n=3. **b)** Immunohistochemical analysis for Ki67 and cleaved caspase-3 of the lung in rats receiving control, NP-control or NP-rapamycin patches at day 0 (upper row), day 7 (middle row) or day 30 (lower row); yellow arrows show representative positive cells; scale bar, 50 µm; n=3. Yellow arrows show representative positive cells; scale bar, 50 µm; n=3. **c)** Bar graph shows the proliferation index; n=3; two-way ANOVA, group, p=0.0742; time, p=0.4306. **d)** Bar graph shows the apoptosis index; n=3; two-way ANOVA, group, p=0.1433; time, p=0.8415.

Supplemental Figure 4: Influence of NP-rapamycin on rat liver, kidney and spleen. **a)** Time course showing immunohistochemical analysis for Ki67 and cleaved caspase-3 of the liver, kidney and spleen in rats receiving control, NP-control or NP-rapamycin patches at day 0 (control), day 7 or day 30 ; scale bar, 100 µm; n=3. **b-g)** Bar graph shows the proliferation and apoptosis index in rat liver, kidney and spleen in rats

receiving control, NP-control or NP-rapamycin patches at day 0 (control), day 7 or day 30; n=3.

Supplemental Figure 5: Full-size Western blots. Western blots shown in Figures 2, 4a and 4d are presented in uncropped full-size format.

supplemental figure 1

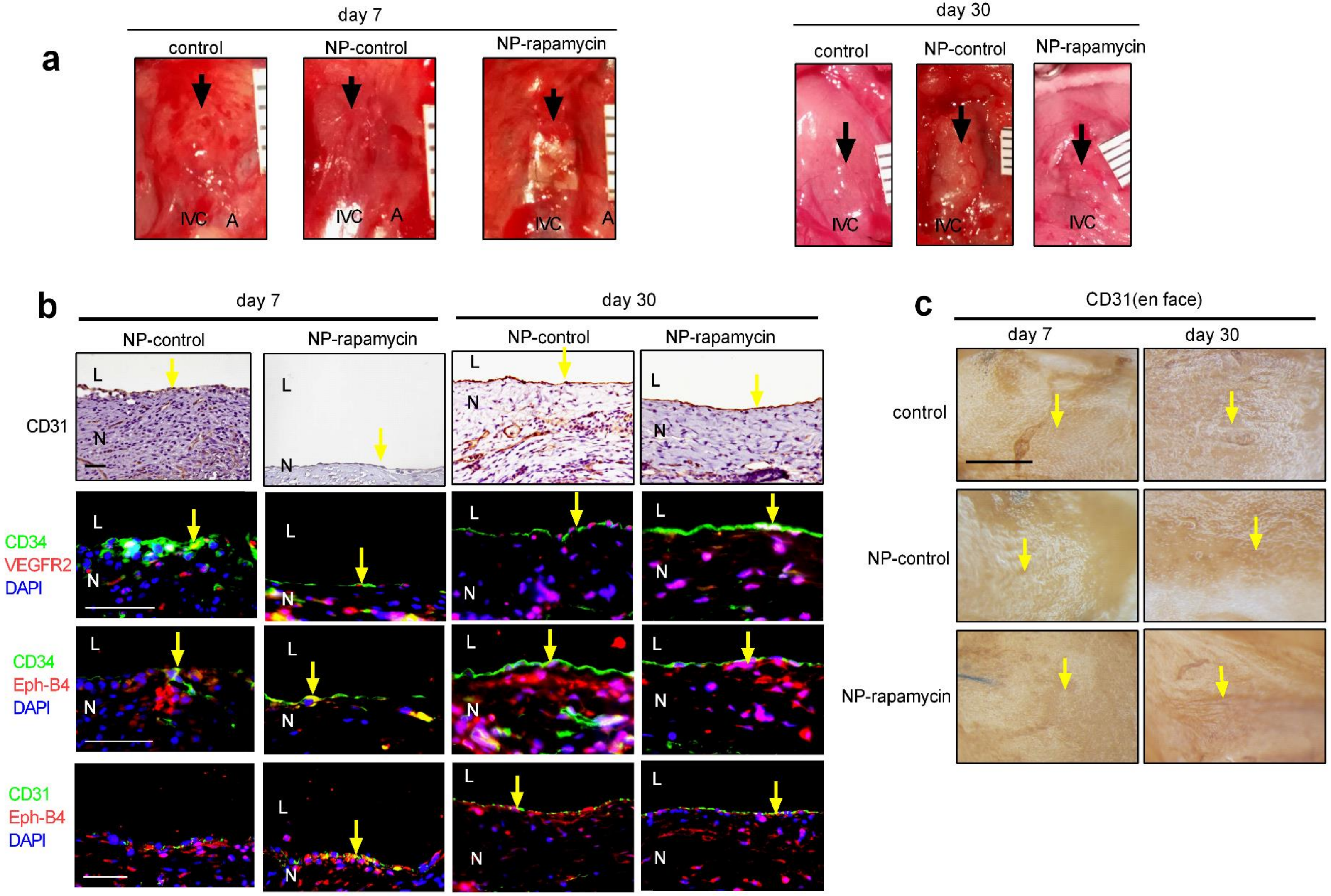

supplemental figure 2

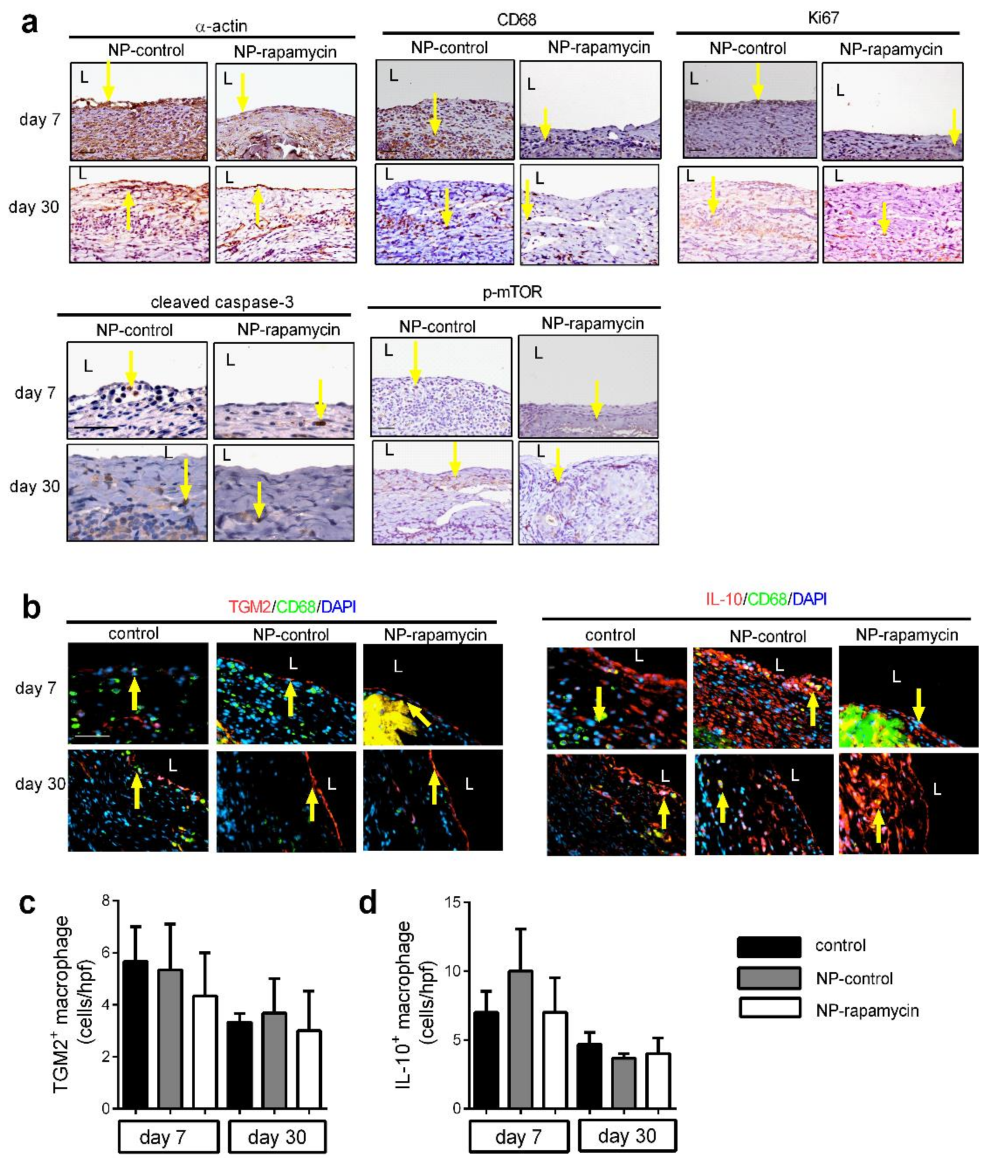

supplemental figure 3

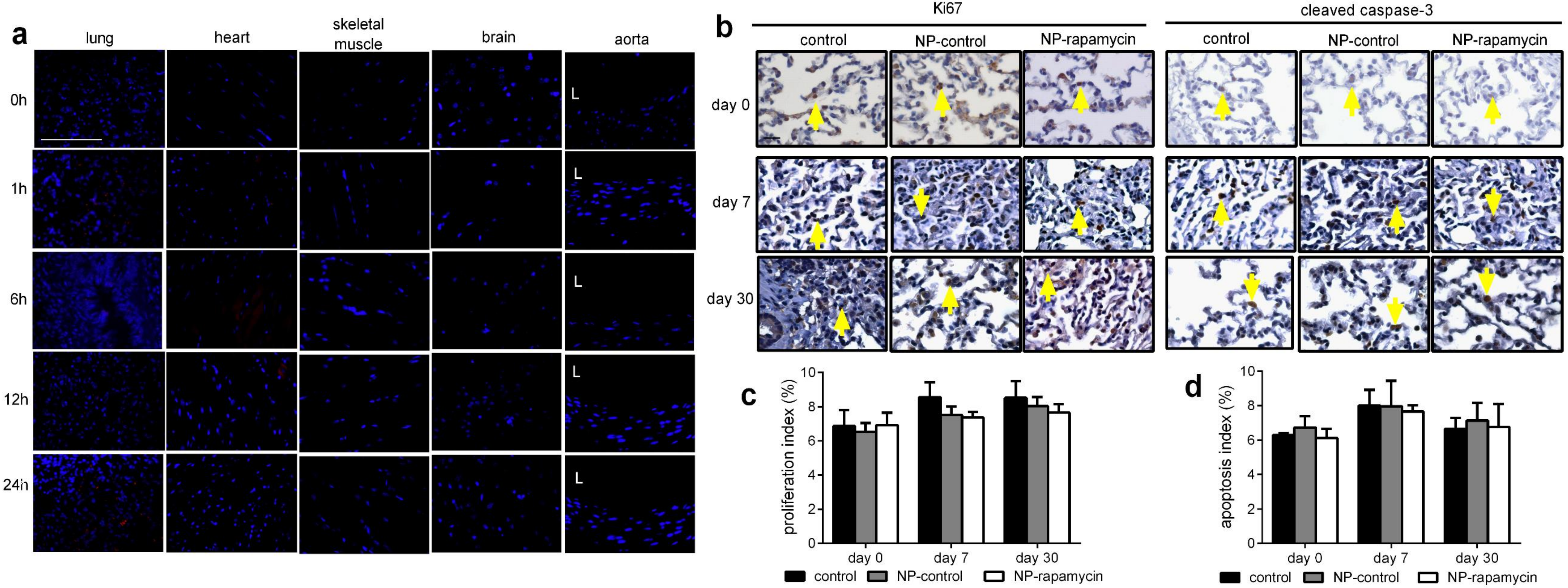

supplemental figure 4

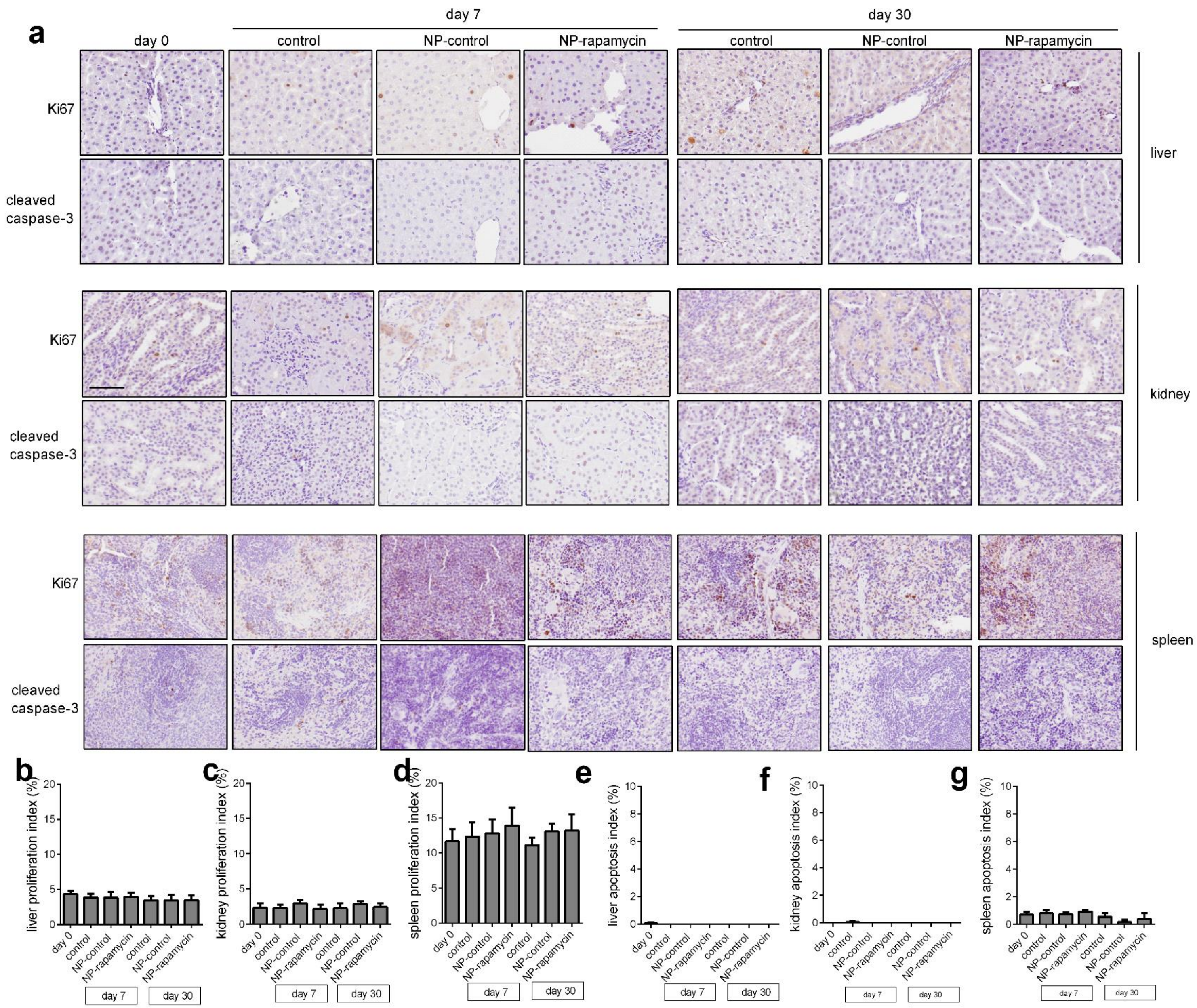

Supplemental Figure 5

Figure 2

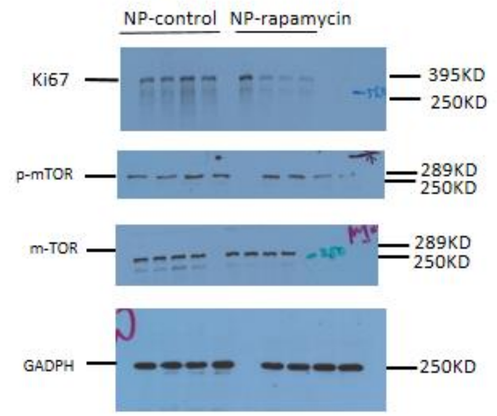

Figure 4a

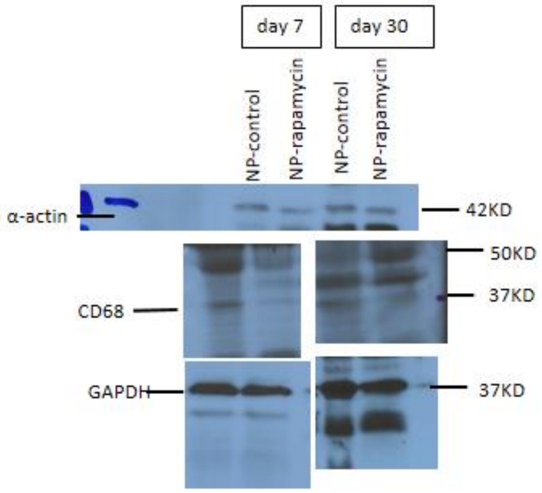

Figure 4d

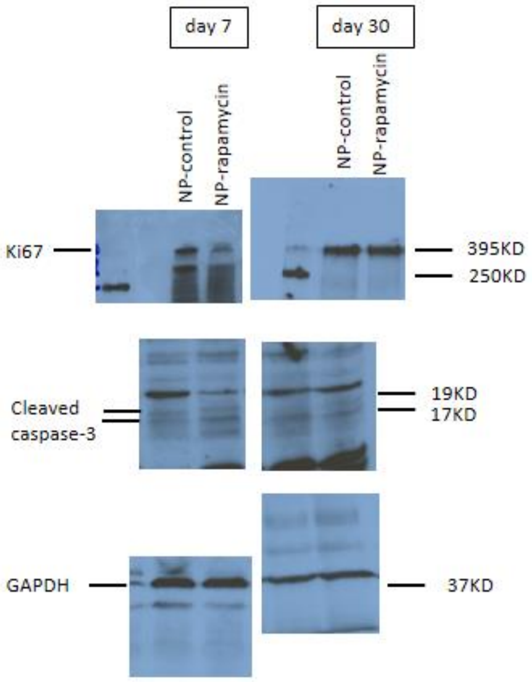

Supplement: Supplementary Figures [file srep40142-s1.pdf]
